# Community-Based Strategies to Improve Health-Related Outcomes in People Living With Hypertension in Low- and Middle-Income Countries: A Systematic Review and Meta-Analysis

**DOI:** 10.5334/gh.1329

**Published:** 2024-06-12

**Authors:** Solomon Nyame, Daniel Boateng, Pauline Heeres, Joyce Gyamfi, Lebo F. Gafane-Matemane, John Amoah, Juliet Iwelunmor, Gbenga Ogedegbe, Diederick Grobbee, Kwaku Poku Asante, Kerstin Klipstein-Grobusch

**Affiliations:** 1 Julius Global Health, Julius Center for Health Sciences and Primary Care, University Medical Center, Utrecht University, Utrecht, The Netherlands; 2 Kintampo Health Research Centre, Ghana; 3 Department of Global Health, New York University School of Global Public Health, New York University, New York, NY, USA; 4 Hypertension in Africa Research Team (HART), North-West University, South Africa; 5 MRC Research Unit for Hypertension and Cardiovascular Disease, North-West University, North-West Province, South Africa; 6 Saint Louis University College for Public Health and Social Justice, St. Louis, MO, USA; 7 Institute for Excellence in Health Equity, NYU Langone Health, New York, NY, USA; 8 Division of Epidemiology and Biostatistics, School of Public Health, Faculty of Health Sciences, University of the Witwatersrand, Johannesburg, South Africa

**Keywords:** hypertension, community-based strategies, low middle income countries, and blood pressure control, CRD42020194081

## Abstract

**Background:**

Individuals living with hypertension are at an increased risk of cardiovascular- and cerebrovascular-related outcomes. Interventions implemented at the community level to improve hypertension control are considered useful to prevent cardiovascular and cerebrovascular events; however, systematic evaluation of such community level interventions among patients living in low- and middle-income countries (LMICs) is scarce.

**Methods:**

Nine databases were searched for randomized controlled trials (RCTs) and cluster randomized control trials (cRCTs) implementing community level interventions in adults with hypertension in LMICs. Studies were included based on explicit focus on blood pressure control. Quality assessment was done using the Revised Cochrane Risk of Bias tool for randomized trials (ROBS 2). Results were presented according to the Preferred Reporting Items for Systematic Reviews and Meta-Analyses (PRISMA) checklist. Fixed-effect meta-analyses were conducted for studies that reported continuous outcome measures.

**Results:**

We identified and screened 7125 articles. Eighteen studies, 7 RCTs and 11 cRCTs were included in the analysis. The overall summary effect of blood pressure control was significant, risk ratio = 1.48 (95%CI = 1.40–1.57, n = 12). Risk ratio for RCTs was 1.68 (95%CI = 1.40–2.01, n = 5), for cRCTs risk ratio = 1.46 (95%CI = 1.32–1.61, n = 7). For studies that reported individual data for the multicomponent interventions, the risk ratio was 1.27 (95% CI = 1.04–1.54, n = 3).

**Discussion:**

Community-based strategies are relevant in addressing the burden of hypertension in LMICs. Community-based interventions can help decentralize hypertension care in LMIC and address the access to care gap without diminishing the quality of hypertension control.

## Introduction

Hypertension affects one billion people globally and is a major risk factor for cardiovascular diseases [1]. Currently, it is estimated that high blood pressure (BP) is related to the deaths of more than 10 million people every year [2]. Estimates suggest that by 2025, the number of adults living with hypertension will increase to approximately 1.56 billion, with more than two-thirds living in low- and middle-income countries (LMICs) [2]. Individuals living with hypertension are at an increased risk of cardiovascular- and cerebrovascular-related mortality [3].

Hypertension management is critical to the prevention of cardiovascular and cerebrovascular events. However, management in LMICs is sub-optimal because of poor access to care, lack of awareness, limited availability of medications [4], and shortage of physicians including at the primary care level [5,6,7]. As a result, there are still significant gaps in managing hypertension particularly in LMICs [8], emphasizing the need to effectively identify individuals with hypertension, and encourage effective disease management measures at the community level. Interventions to mitigate the looming hypertension crisis in LMIC need a strong community component whilst concurrently addressing access to care and quality of care issues.

Community-based intervention is a multi-faceted technique that combines individual and environmental change strategies across multiple settings aiming to prevent dysfunction and to promote well-being among population groups in a defined local community [9]. This includes health education, outreach services, self-management, and home-based care, which have emerged as practical approaches to addressing the critical gap in access to care [10,11,12]. Literature suggests community-based interventions focused on hypertension are cost-effective and promote positive health outcomes [9,10,11,12,13]. Evidence from studies conducted in LMICs suggest improved hypertension control for patients receiving community health worker (CHW) home visits [10,11,12] and reduction in BP related to a CHW-led chronic disease programme [10,11,12] and use of mobile technology [10,11,12]. Thus, it is essential to comprehensively document interventions implemented at the community level for hypertension control in LMICs to complement previous evidence on community-based interventions for cardiovascular diseases prevention in LMIC [13]. Also, what remains unknown is the synthesis of how these community-based strategies impact blood pressure control in LMICs. The objective of this systematic review was to evaluate community level interventions targeting improvement in hypertension control among patients LMICs.

## Methods

We conducted the systematic review and meta-analysis in accordance with the Preferred Reporting Items for Systematic Reviews and Meta-Analyses (PRISMA) checklist for the PROSPERO registered protocol (CRD42020194081).

### Inclusion criteria

We included studies on community-based interventions for hypertension control for adults aged 18 years and older with hypertension. Hypertension was defined as average clinic systolic BP (SBP) ≥ 140 mm Hg or diastolic BP (DBP) ≥ 90 mmHg following JNC-7 guidelines [14]. The included studies were mostly conducted at the community level, population level, health care provider level, community level, and at health care facilities in LMICs. We evaluated studies published between January 2000–July 30, 2023.

### Exclusion criteria

Studies that are not RCTs and cRCTs were excluded. Finally, we excluded studies conducted at the population level, health care provider level, community level, and at health care facilities not located within LMICs.

### Information sources

We searched for articles published in English from January 2000 until July 30, 2023. The databases searched are PubMed, Scopus, Web of Science, Global Health (CABI), PsycInfo, CINAHL, MEDLINE, Cochrane Library, and EMBASE databases. References of relevant articles were also screened.

### Search strategy

The search strategy was based on MeSH terms ‘low- and middle-income countries’, ‘developing countries’, ‘community-based strategy’, ‘health education’, and ‘blood pressure’ (see Appendix 1).

### Study selection process

Two researchers (SN and PH) independently conducted the initial screening of study titles and abstracts to identify relevant articles using Rayyan [15,16]. Subsequently, relevant studies were retrieved with full texts for further assessment. Based on the inclusion criteria SN and PH independently selected the eligible studies. Any discrepant selection was discussed and resolved with DB. The following types of studies were included: randomized controlled trials (RCTs), cluster randomized controlled trials (cRCTs), and conducted in countries referred to as low-middle-income country (by the World Bank at the time of the literature search). Qualitative studies and studies that measured health outcomes through self-report were excluded because these studies do not provide quantifiable and valid measures for hypertension control data. The search was limited to journal articles (published in English) from 2000 till 2023 since implementation of community level intervention for hypertension became prominent within these periods.

### Data collection process, data items and risk of bias assessment

A data extraction form was designed on a validated Research Electronic Data Capture (REDCap) project as a web-based application [17]. Two researchers (SN and PH) independently entered the data based on the following information: study design, country and setting, sample size, main outcome, and secondary outcomes. Three researchers (SN, DB and LFG) conducted the quality assessment using the Revised Cochrane Risk of Bias tool for randomized trials (ROBS 2) [18,19]. Bias was assessed on five domains: randomization process, deviations from the intended intervention, missing outcome data, measurement of outcome and the selection of the reported result. Bias risk was assigned as either one of three levels (low/high/or some concerns). Some concerns were selected for studies where the risk of bias was unclear based on the reported information.

### Synthesis methods and effect measure

We conducted a meta-analysis for the effect of interventions on blood pressure control. A random effects model was used due to the heterogeneity of the varying studies in terms of varying interventions, outcome measures, study population and non-stratification of the study outcome. Subsequently, sub-group analysis was done for the two types of studies (RCTs and cRCTs). We also undertook a sub-group analysis for studies that reported data for the various components of the interventions. For such studies (n = 3), the N for the control group was divided by the number of interventions [20]. All meta-analyses were conducted using RevManWeb (Cochrane collaboration). Authors observed that most of the studies combined the effect of multicomponent interventions. Heterogeneity was assessed using the Cochrane’s Q and degree of inconsistency (I^2^) [21]. All analyses were considered statistically significant at the two-sided 5% level (*p* < 0.05). We could not estimate the effect of the interventions on changes in BP due to insufficient data reporting in most of the studies. Findings of the remaining studies were presented in a narrative format. Where there were multiple measurements at different time points, the team considered estimates for the endline assessment since these were considered clinically significant, as suggested by the Cochrane Handbook Chapter 3, Section 3.2.4.3 [22].

## Results

### Study selection

We derived 7125 articles from our search in PubMed, Embase, Scopus, Web of Science, Global Health (CABI), PsycInfo, MEDLINE, Cochrane Library, and CINAHL. We imported the citations and full texts for review in Rayyan after removal of duplicates. Of the unique 6851 citations reviewed, 6722 citations were excluded after screening the title and abstract. The remaining 129 were assessed by reviewing the full text. During the full text review, a more in-depth evaluation of each article was performed, after which 110 records were excluded and 19 included for analysis. Figure 2 highlight this breakdown.

### Study characteristics

**Figure 1:**
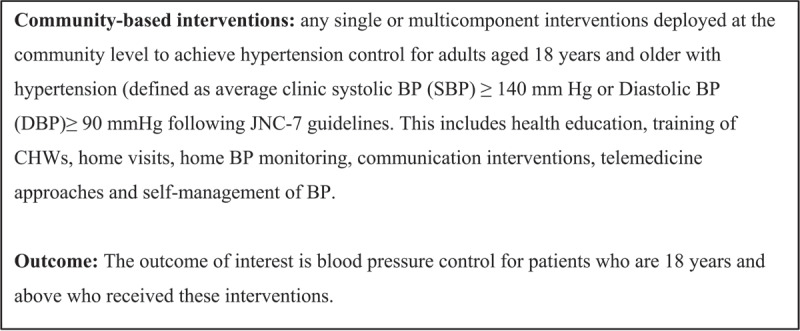
Description of key terminologies.

Details on the study selection are provided in Figure 1. The 19 included studies were conducted at the population level, health care provider level, community level, and at health care facilities in LMICs. The characteristics of all the studies included in this systematic review are presented in Table 1. The studies included were RCTs (n = 9) and cRCTs (n = 10). Seven of the studies were conducted in rural settings whereas five of the studies were conducted in urban settings, six studies did not provide setting information. The studies were conducted in Argentina [22], China [23,24,25,26], India [27,28,29,30], Iran [31], Kenya [32], Nepal [33,34,35], Nigeria [36], Pakistan [37], and Vietnam [38]. Two of the studies were conducted in multiple countries: Kenya and Uganda [39] as well as Bangladesh, Pakistan and Sri Lanka [40]. The sample size of the studies included in this review ranged from 50 to 3556.

**Figure 2:**
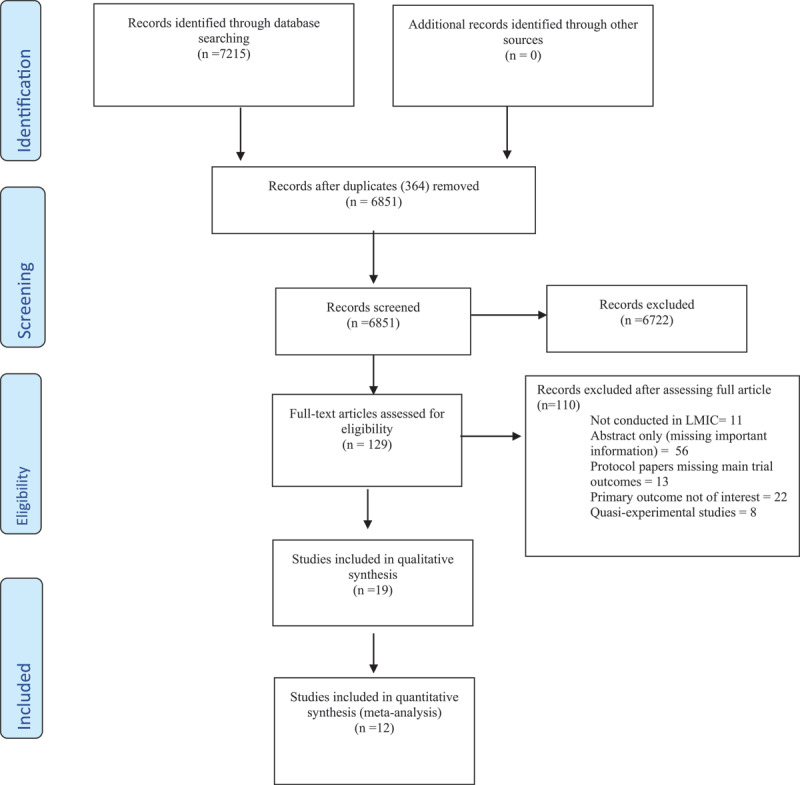
Description of the study selection processes.

**Table 1:** Study characteristics of studies assessing community-based strategies to improve hypertension outcomes.


AUTHORS, (YEAR), COUNTRY	STUDY DESIGN	SETTING	SAMPLE SIZE	STUDY PARTICIPANTS				MAIN OUTCOME	SECONDARY OUTCOME	RISK OF BIAS

				INTERVENTION		CONTROL/COMPARATOR				
	
				NO. OF PARTICIPANTS	MEAN AGE	NO. OF PARTICIPANTS	MEAN AGE			

Jafar et al., (2009) [38], Pakistan	Cluster-randomized, controlled trial	NA	1341	1015 (3 different groups of intervention)	53.8–55.3	326	53.3	Change in systolic blood pressure	The proportion of people with controlled hypertension (BP < 140/90 mm Hg)	Some concern

Nguyen et al., (2018) [39], Vietnam	Cluster-randomized controlled feasibility trial	Rural	160	80	66	80	66.9	Patient’s levels of Systolic and diastolic blood pressure	The proportion of patients with controlled hypertension	Some concern

Nuepane et al., (2018) [34], Nepal	Open-label, cluster-randomized trial	Urban	435	255	50.1	180	50.3	Mean systolic blood pressure at 1 year	Change in mean diastolic blood pressure	Low

Vedanthan et al., (2019) [33], Kenya	Cluster-randomized trial	Rural	1460	500 and 469	53.7, 54.3	491	54.6	Linkage to care	Change in SBP	High

Li et al., (2019) [24], China	Cluster-randomized control trial	NA	462	186	61.7	276	61.3	SBP change between baseline and follow-up	DBP, BP control	High

Gamage et al., (2020) [31], India	Cluster-randomized controlled trial	Rural	1734	637	56.6	1097	56.9	The proportion of people with controlled hypertension (BP < 140/90 mm Hg)	Change in SBP and DBP	High

Jafar et al., (2020) [41], Bangladesh, Pakistan and Sri Lanka	Cluster-randomized, controlled trial	Rural	2645	1330	58.5	1315	59.0	Mean change in systolic Blood pressure from baseline to 24 months	Diastolic blood pressure and % of participants with blood pressure control	Some concern

Khanal et al., (2021) [35], Nepal	Cluster-Randomized Controlled Trial	Rural	125	63	56.6	62	56.6	Normalized SBP	Controlled DBP, mean difference of SBP, waist circumference and Knowledge score	Some concern

Suseela et al., (2022) [30], India	Cluster-randomized Controlled pragmatic Trial	Urban slums	1952	968	56.8	984	55.7	Change in mean SBP	Proportion of patients on antihypertensive medication, change in self-reported medication adherence scores, change in BMI, self-reported tobacco uses and per capita monthly consumption of salt	Some concern

Thapa et al., (2023) [36], Nepal	Open-label, cluster-randomized trial	Rural	1638	939	45.4	699	45.3	Mean change SBP	Mean change in DBP, the proportion of participants with new diagnosis of hypertension, the proportion of those who were aware of their hypertension status, Change in use of antihypertensive medication use.	Some concern

Adeyemo et al., (2013) [37], Nigeria	Randomized controlled trial	Rural and Urban	668	668	63	-	-	Pill count and biological assay with a urinary riboflavin tracer	Mean BP level	High

Lu et al., (2015) [26], China	Randomized, non-blinded trial	NA	360	233	53.8	114	55.9	Change in the proportion of subjects with normalized BP	-	Some concern

He et al., (2017) [23], Argentina	Randomized clinical trial	Urban	1432	1432	55.8	-	-	Differences between the intervention and control groups in mean systolic and diastolic BP changes	Proportion of patients who had controlled hypertension	Some concern

Qi, Qiu, and Zhang., (2017) [25], China	Prospective, double-blind, randomized study	NA	1183	533	63.5	499	64.5	Reduction in systolic and diastolic BP	-	Some concern

Pan et al., (2018) [27], China	Randomized control trial	Urban	107	52	56.6	55	57.8	Average changes in blood pressure	Post-interventional control rate	Some concern

Sany et al., (2018) [32], Iran	Randomized controlled trial	NA	240	240	54.8	-	-	Changes in SBP and DBP	Medication Adherence	Some concern

Sheilini et al., (2019) [29], India	Randomized controlled study	NA	160	64	-	60	-	Medication adherence level	Changes in SBP and DBP	Low

Khetan et al., (2019) [28], India	Randomized controlled trial	Urban	3556	736	52.1	506	51.7	Change in SBP from visit 1 to post-intervention	Mean reduction in diastolic blood pressure	Some concern

Hickey et al., (2022) [40], Kenya and Uganda	Randomized controlled trial	Rural	199	99	56	100	56	Linkage to care	Blood pressure control	Some concern


#### Overall risk of bias assessment of intervention studies

**Figure 3:**
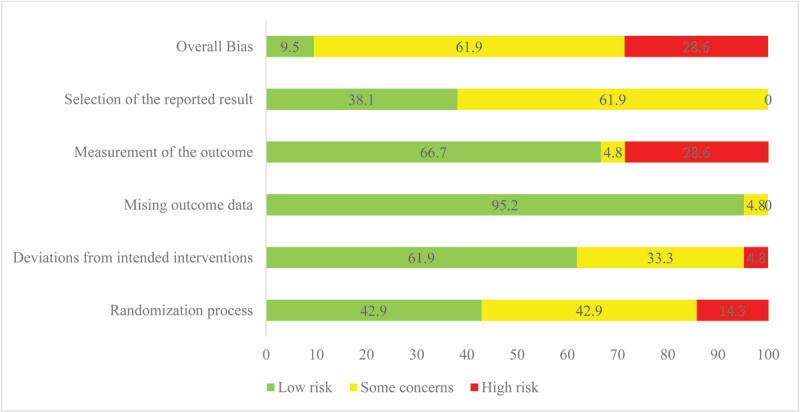
Overall Risk of bias.

Table 1 highlights the risk of bias of the individual studies. Figure 3 shows the overall risk of bias. For the cRCT, three studies [23,30,32] were ‘high risk’, three studies had ‘some concern’ and one study’s assessment indicated ‘low risk of bias’. For the cRCT studies, six studies were of ‘some concerns’, one study was identified to be ‘high risk’ as well as one study was identified to be ‘low risk’ for the domains considered. A summary of the overall risk assessment for the RCT and cRCTs is shown in Figure 5, whereas the detailed quality assessment is shown on Supplementary Figures 1 and 2, respectively.

The RCTs generally performed well in their risk of bias for measurement of outcome (77.8%), missing outcome data (100%), and deviation from intended interventions (66.7%). The majority (66.7%) had some concerns in selection of the reported result and 11.1% had high risk of bias in measurement of outcome and randomization process (Supplementary Figure 1). In the detailed presentation in Supplementary Figure 1, Sheilini et al. [28] had a low risk of bias in all domains. Adeyemo et al. [36] had high risk of bias in the measurement of outcomes, some concerns in the randomization process and selection of the reported results and an overall high risk of bias.

Overall, only a small proportion of cRCTs reported low risk of bias (14.3%). The majority reported some concerns (85.7%). Several studies displayed low risk of bias for the deviation from intended interventions (57.1%), missing outcome data (100%), measurement of outcome (100%) and selection of the reported result (85.7%) domains. All the cRCT studies had some concerns with the randomization process. Neupane et al. [33] had low risk of bias in all domains except for the randomization process, whereas Gamage et al. [30] had low risk of bias in two domains and some concerns in three domains as shown in Supplementary Figure 2.

#### Intervention characteristics and effect on BP

Table 2 highlights the intervention characteristics of the included studies. The intervention settings were mostly community-based; however, three of the studies were both facility- and community-based [28,36,38]. The minimum duration for the intervention delivery was two months whereas the maximum duration was 60 months. One study did not have any information on the duration of the intervention delivery. The intervention components included health education, training of health workers, telemedicine approaches, home visits and BP monitoring, communication skills intervention, and self-management. Fifteen of the studies used multicomponent interventions whereas four of the studies used a single component intervention, mainly focusing on health education (health promotion), training of community health workers and home blood pressure monitoring. The intervention was mostly compared to usual/routine care.

**Table 2:** Intervention characteristics of studies assessing community-based strategies to improve hypertension outcomes.


AUTHORS, (YEAR), COUNTRY	INTERVENTION CHARACTERISTICS				CONTROL GROUP/COMPARATOR DESCRIPTION

	INTERVENTION SETTING	DURATION OF INTERVENTION	INDIVIDUALS DELIVERING INTERVENTION	INTERVENTION COMPONENTS	

Jafar et al., (2009) [37], Pakistan	Community-based	24 months	Community health workers/general practitioners	Family-based home health education (HHE) from trained lay health workers every 3 months. Annual training of General Practitioners in hypertension management.	Usual Care

Adeyemo et al., (2013) [36], Nigeria	Facility-based and community based	6 months	Nurses	Multicomponent Intervention: Clinic-based treatment; provision of free antihypertensive medications; and Reimbursement of transportation costs for a monthly clinic visit	Home visit and routine clinic care

Lu et al., (2015) [25], China	Community-based	24 months	General practitioners	Multicomponent Intervention: Self-reading learning; regular lecture and Interactive workshop	Regular lecture and interactive lecture

He et al., (2017) [22], Argentina	Community-based	18 months	Community health worker	Multicomponent Intervention: Community health worker-led home intervention (health coaching, home BP monitoring, and BP audit and feedback), a physician intervention, and a text-messaging intervention over 18 months	Usual care with no intervention

Qi, Qiu, and Zhang., (2017) [24], China	Community-based	60 months	Patients/participants	Non-multicomponent intervention: Home Blood Pressure Monitoring	Measured and recorded BP in the community

Nguyen et al., (2018) [38], Vietnam	Facility-based and community based	12 months	Community health worker	Multicomponent Intervention: Storytelling intervention (DVDs); didactic “Learn More” content (DVDs)	Received a single DVD

Neupane et al., (2018) [33], Nepal	Community-based	12 months	Female community health volunteers (FCHVs)	Multicomponent Intervention: Home visits every 4 months for lifestyle counseling and blood pressure monitoring.	Usual care about current practices for HTN management at the community level.

Pan et al., (2018) [26], China	Community-based	12 months	GP, a hypertension specialist, a general nurse, and an information manager.	Non-multicomponent intervention: Home telemonitoring for blood pressure	Usual care (no automated BP device for home monitoring)

Sany et al., (2018) [31], Iran	Community-based	NA	Health providers	Non-multicomponent intervention: communication skills intervention	Usual Care (no definition of usual care)

Sheilini et al., (2019) [28], India	Facility-based and community based	6 months	Nurses	Multicomponent Intervention: Individualized teaching on mediation adherence and healthy lifestyle practices; information leaflet on medication adherence and healthy lifestyle practices; weekly medication-reminder boxes; and telephonic reminder for follow-up.	Routine care

Khetan et al., (2019) [28], India	Community-based	24 months	Community health worker	Multicomponent Intervention: Training of Community Health Workers; Community Health Worker led home-based counseling; Physician examination	Usual Care in the community

Vedanthan et al., (2019) [32], Kenya	Community-based	15 months	Community health workers	Multicomponent Intervention: “Paper-based” (tailored behavioral communication, using paper-based tools); and “smartphone” (tailored behavioral communication, using smartphone technology).	Usual Care (Standard Training)

Li et al., (2019) [23], China	Community-based	6 months	Family doctors	Multicomponent Intervention: Health education; health promotion, group chat, and BP monitoring	Usual community health care services (Health lectures and one chronic disease follow-up)

Gamage et al., (2020) [30], India	Community-based	3 months	Community health workers	Multicomponent Intervention: Monitoring of BP, education about hypertension, and support for a healthy lifestyle change	Usual care

Jafar et al., (2020) [40], Bangladesh, Pakistan and Sri Lanka	Community-based	24 months	Community health workers	Multicomponent Intervention: Blood pressure monitoring and the use of a checklist to guide monitoring and referral to Physicians.	Usual care (Existing services in the community and routine home visits)

Khanal et al., (2021) [34], Nepal	Community-based	6 months	Medical school graduate and registered nurses	Multicomponent Intervention: Four health education sessions and home visit and usual care	Usual care

Suseela et al., (2022) [29], India	Community-based	6 months	Women’s self-help groups members	Multicomponent Intervention: Assistance in daily hypertension management, social and emotional support to encourage healthy behaviours and referral to the primary health care system	Standard care

Hickey et al., (2022) [39], Kenya and Uganda	Community-based	3 months	Community health workers	Multicomponent Intervention: Linkage incentive - provision of transportation vouchers (worth $5.00) and follow up phone calls	Usual care (No linkage incentive and No follow up phone calls)

Thapa et al., (2023) [35], Nepal	Community-based	12 months	Female community health volunteers	Multicomponent Intervention: Home visits lifestyle counselling and blood pressure measurement	Usual care


Table 3 presents the key findings (main outcome and secondary outcome) for the studies included in this systematic review. Overall, the studies reported some form of improvement in the outcomes of interest such as linkage to care, improvement in systolic and diastolic blood pressure and medication adherence. As highlighted in Table 3, there were various definitions of the main primary outcomes as well as secondary outcomes for the studies used in this review. A total of six studies reported change in systolic BP as the main outcome [23,26,27,33,37,40]. Also, four of the studies reported changes in both systolic BP and diastolic BP as the main outcome [22,24,31,38]. Three of the studies reported mean medication adherence as the main outcome [28]. Two studies reported changes in BP control as the main outcome [25,30]. The remaining studies each reported pill count, as well as linkage to care [32]. Regarding the secondary outcomes, four of the studies reported on control of BP [22,26,37,38]. Two studies reported on changes in both systolic and diastolic BP as the secondary outcomes [35,38]. Two studies reported on diastolic BP as well as BP control as the secondary outcomes [23,40]. One study reported on mean BP level [41] as well as medication adherence level/score [31]. The remaining studies reported on quality of life as well as change in SBP [32].

**Table 3:** Key findings of studies assessing community-based strategies to improve hypertension outcomes.


AUTHORS, (YEAR), COUNTRY	KEY FINDINGS			

	MAIN OUTCOME		SECONDARY OUTCOME	
	
	INTERVENTION	CONTROL/COMPARATOR	INTERVENTION	CONTROL/COMPARATOR

Jafar et al., (2009) [37], Pakistan	Mean systolic blood pressure fell by 9.0 mm Hg in the intervention group	Mean systolic blood pressure fell by 3.9 mm Hg in the control group	Blood-pressure control (<140/90 mm Hg) was achieved in 53.2% of those in the intervention group	Blood-pressure control (<140/90 mm Hg) was 43.7% of those in the control group (relative risk, 1.22; 95% CI, 1.10 to 1.35).

Adeyemo et al., (2013) [36], Nigeria	~77% of participants took > 98% of prescribed pills	–	Hypertension control (BP < 140/90 mmHg) was achieved in ~66% of participants	–

Lu et al., (2015) [25], China	Normalized BP increased significantly (from 41.2% to 63.2%, p < 0.001) increased more substantially in Group 3 (from 40.2% to 86.3%, p < 0.001)	–	–	–

He et al., (2017) [22], Argentina	Systolic BP reduction from baseline to month 18 was 19.3 mmHg (95%CI, 17.9–20.8 mmHg)	12.7 mmHg (95%CI, 11.3–14.2 mmHg) for the usual care group	Patients with controlled hypertension increased from 17.0% (baseline) to 72.9% at 18 months in the intervention group	Increased from 17.6% (baseline) to 52.2% at 18 months in the usual care group

Qi, Qiu, and Zhang., (2017) [24], China	Decrease in the systolic pressure by (4.3 ± 3.2) mmHg (P < 0.05)	Decrease in the systolic pressure by (3.9 ± 3.1) mmHg (P < 0.05)	Decrease in diastolic pressure by (3.5 ± 2.5) mmHg (P < 0.05).	Diastolic pressure decreased by (3.0 ± 2.5) mmHg (P < 0.05).

Pan et al., (2017) [26], China	Reduction in blood pressure systolic blood pressure was 16.4 (12.3–18.3)	Reduction in blood pressure systolic blood pressure was 9.8 (6.2–13.5)	Blood pressure control was achieved for 63.6%	Blood pressure control was achieved for 41.80

Sany et al., (2018) [31], Iran	Reduction SBP from 145.6 ± 13.8 to 124.2 ± 7.2 Reduction DBP from 91.50 ± 9.6 to 78.16 ± 6.3	Reduction SBP from 146.1 ± 15.0 to 143.8 ± 13.0 The was reduction DBP from 89.53 ± 9.6 to 87.16 ± 10.0	Increased from 84.08 ± 9.09 to 102.22 ± 12.13	Increased from 82.20 ± 10.0 to 83.18 ± 8.0

Nguyen et al., (2018) [38], Vietnam	Patient’s mean systolic blood pressure declined by 10.7 mmHg (95% CI: 6.5–14.9 mmHg) in the storytelling intervention	Patient’s mean systolic blood pressure declined by 5.8 mmHg (95% CI: 1.6–10.0 mmHg) in the didactic intervention group.	Proportion of patients with controlled hypertension was 31.4 (18.4–44.3) for the storytelling Group	Proportion of patients with controlled hypertension was 20.5 (7.2–33.7) for the Didactic Group

Neupane et al., (2018) [33], Nepal	Change in mean SBP = –6·47	Change in mean SBP = –2.85	Change in mean DBP = –2·90	Change in mean DBP = –1.11

Li et al., (2019) [23], China	Mean difference in SBP from baseline was –5.3 (–8.2, –2.4).	The mean difference in SBP from baseline was 1.6 (–1.2, 4.5)	The mean difference in DBP from baseline was –1.1 (–2.7, 0.6)	Mean difference in DBP from baseline was 2.0 (0.6, 3.7)

Vedanthan et al., (2019) [32], Kenya	Linkage to care for the paper-based and Smartphone was 43% and 54%	Linkage to care for the usual care group was 50%	Change in SBP for the Paper-based and smartphone was –8.4 ± 24.0 and –13.1 ± 20.5 respectively	Change in SBP for the usual care group was –9.7 ± 25.1

Sheilini et al., (2019) [28], India	At 6 months the mean medication adherence was 8.00 ± 0.00 compared to the mean medication adherence at baseline of 5.59 ± 0.49	At 6 months the mean medication adherence score was 7.70 ± 0.72 compared to the mean medication adherence score at baseline of 5.93 ± 0.44	SBP post 6-months was 153.28 ± 12.85 compared to a baseline SBP of 154.34 ± 10.34. DBP post 6-months was 84.96 ± 6.89 compared to a baseline DBP of 86.28 ± 7.01.	SBP post 6-months was 154.83 ± 11.57 compared to a baseline SBP of 154.66 ± 11.26. DBP post 6-months was 87.30 ± 7.99 compared to a baseline DBP of 85.73 ± 6.58.

Khetan et al., (2019) [27], India	Mean ± SD change in systolic blood pressure at 2 years was –12.2 ± 19.5 mm Hg	Mean ± SD change in systolic blood pressure at 2 years was –6.4 ± 26.1 mm Hg	Mean ± SD change in diastolic blood pressure at 2 years was –5.1 ± 13.5	Mean ± SD change in diastolic blood pressure at 2 years was –3.0 ± 14.7

Gamage et al., (2020) [30], India	Control of BP improved from baseline to follow-up from 49.5% to 69.7% of the intervention group	Control of BP improved from baseline to follow-up from 52.2% to 61.7% of the usual care group	Decline in systolic BP in the intervention group was 8.2 mmHg. Decline in diastolic BP in the intervention group was (4.2 mm Hg)	Decline in systolic BP in the usual care group was 2.1 mmHg. Decline in diastolic BP in the usual care group was 2.2 mmHg.

Jafar et al., (2020) [40], Bangladesh, Pakistan and Sri Lanka	Mean systolic blood pressure fell by 9.0 mm Hg in the intervention group.	Mean systolic blood pressure fell by 3.9 mm Hg in the control group	The mean diastolic blood pressure fell by –6.07 (–6.85 to –5.29) Blood-pressure control (<140/90 mm Hg) was achieved in 53.2% of the participants in the intervention group	The mean diastolic blood pressure fell by –3.24 (–4.03 to –2.45) Blood-pressure control (<140/90 mmHg) was achieved in 43.7% of the participants in the control group

Khanal et al., (2021) [34], Nepal	Proportion of controlled SBP was 58.3%	Proportion of controlled SBP was 40%	Proportion of controlled DBP was 30% Mean change in SBP was 18.8 mmHg.	Proportion of controlled DBP was 20% Mean change in SBP was 11.2 mmHg

Suseela et al., (2022) [29], India	Mean reduction in SBP was 6.3 mmHg	Mean reduction in SBP was 2.2 mmHg	Percentage change in patient using antihypertensive was 14.2% Change in self-reported medication adherence was 1.44. Change in Self-reported tobacco use was –1.5	Percentage change in patient using antihypertensive 8.8% Change in self-reported medication adherence was 0.58. Change in Self-reported tobacco use was –0.3

Hickey et al., (2022) [39], Kenya and Uganda	Linkage to care was 96%	Linkage to care was 66%	Blood-pressure control (<140/90 mm Hg) was achieved in 51% of the participants in the intervention group	Blood-pressure control (<140/90 mmHg) was achieved in 41% of the participants in the control group

Thapa et al., (2023) [35], Nepal	Increase in mean SBP was 10.4 mmHg	Increase in mean SBP was systolic 6.3 mmHg	Increase in mean DBP was systolic 5.7 mmHg	Increase in mean DBP was systolic 3.2 mmHg


**Figure 4:**
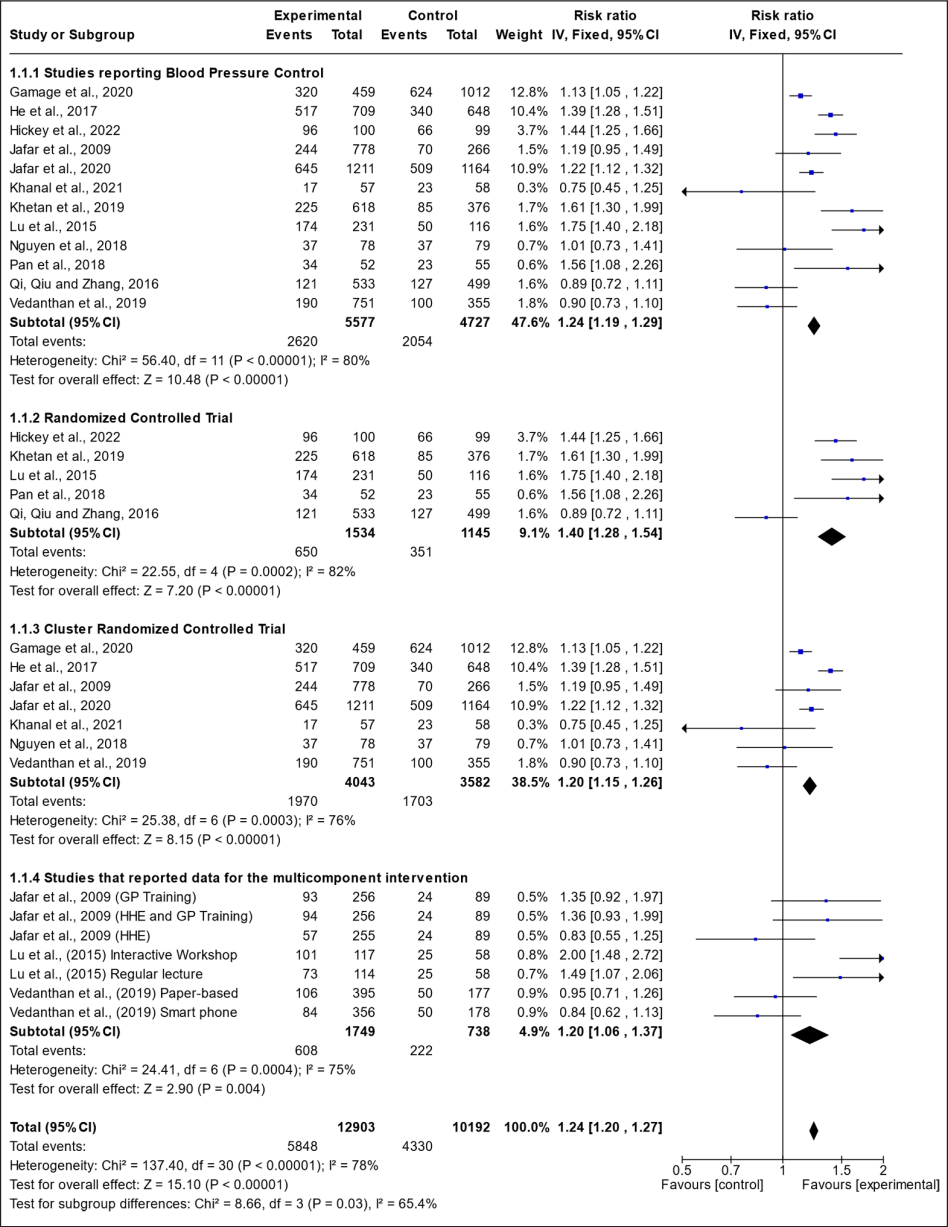
Forest plot for all studies highlighting the effect of the intervention on blood pressure control.

In a meta-analysis of 12 included studies (Figure 4) with n = 12903 participants, the implemented interventions were associated with BP control (RR: 1.24; 95%CI: 1.20–1.27). There was a high degree of heterogeneity as depicted by an I^2^ of 78%. A subgroup analysis highlighted a significant association between community-based interventions and increased BP control (RR: 1.40; 95%CI: 1.28–1.54) among the RCTs (Figure 4). Analysis of the cRCTs also indicated a significant association between community-based interventions and BP control (RR: 1.20; 95%CI: 1.15–1.26) (Figure 4). There was a high degree of heterogeneity for the RCTs as depicted by an I^2^ of 82% whereas this was low for the cRCTs as depicted by an I^2^ of 76%. A sub-group analysis of studies that used CHWs to deliver the intervention showed significant association with BP control, although the heterogeneity was high (RR = 1.54, 95% CI = 1.40–1.69; I^2^ of 81.3%) (Supplementary Figure 3).

**Figure 5:**
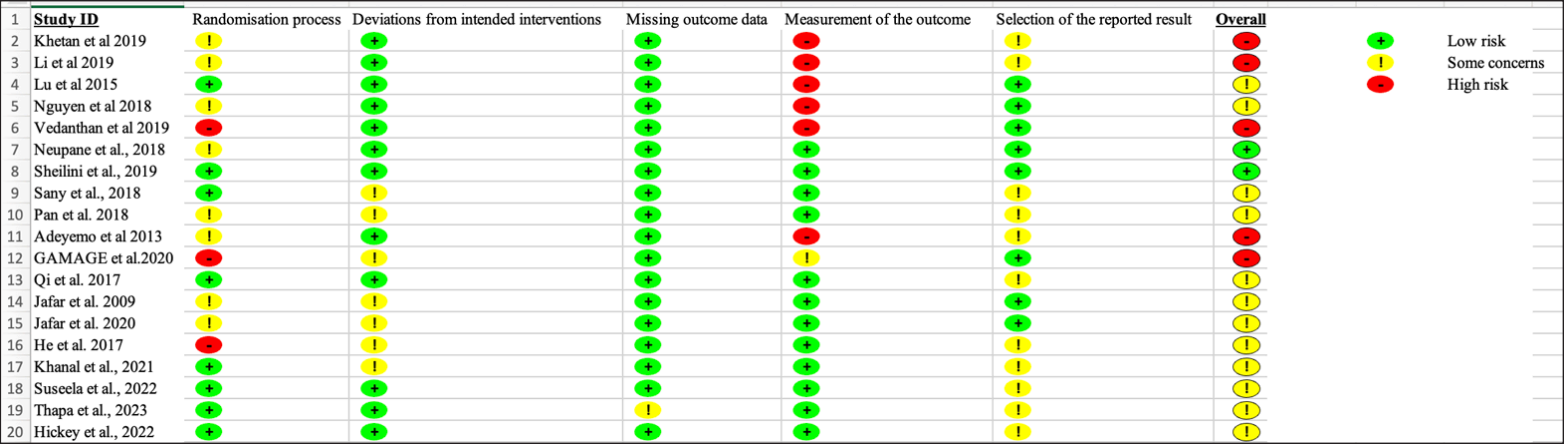
Highlights of the risk of bias domains.

## Discussion

In this review, we assessed the evidence for community-based interventions to address the burden of hypertension as well as the impact of these interventions on BP control. We reviewed 19 studies that used community-based strategies for addressing the burden of hypertension in LMIC. Findings from the meta-analysis showed that community-based interventions significantly led to BP control. The community-based interventions were mostly delivered by CHWs. The interventions from these studies were mostly multicomponent and involved strategies such as health education, training of CHWs, home visits, home BP monitoring, communication interventions, telemedicine approaches and self-management of BP. Results from this review highlight the limited use of technology to address hypertension control at the community level.

Findings from this review and meta-analysis suggest that 12 studies highlighted in these community-based interventions were associated with BP control. Out of these 12 studies, seven were cRCTs and five were RCTs. Reviewing the components of the interventions associated with BP control, we note that home BP monitoring by CHWs was the most used strategy employed at the community level. Agarwal et al. (2011), in their systematic review that sought to quantify the benefit of home BP monitoring on BP reduction, indicated that both systolic and diastolic BP improved with home-based BP monitoring [42]. A total of six of the eight RCT studies used CHWs as the main personnel for delivering the intervention [22,27,30,32,38,40]. A meta-analysis for the studies that used CHWs resulted in significant improvement in BP control. This is an indication that community-based interventions are effective in addressing the suboptimal control of hypertension in LMICs. Also, using community-based interventions can help decentralize hypertension care in LMICs and address the access to care gap in LMICs without diminishing the quality of hypertension control.

There is an increasing focus on using community level resources to address the burden of hypertension as observed in this review. Studies have highlighted the importance of using CHWs in the care of people with hypertension. Brownstein and colleagues note that CHWs deliver culturally relevant and appropriate education, counseling, and social support, and can be trained to provide clinical services such as measuring BP [43,44]. Thus, when adequately trained, CHWs are effective at providing preventative services as well as controlling BP at the community level. Despite this, there are limited guidelines that highlight the use of CHWs for the care of hypertension patients at the community level. The World Health Organization (WHO) HEARTS Technical Package provides pragmatic interventions for strengthening the management of CVDs within the primary health care settings [45]. It stresses the need for health systems to be reoriented and strengthened to respond effectively to the rising burden of CVD with a proactive, community-based, and sustainable patient-centered chronic care system [45]. Therefore, there is the need to develop specific guidelines that will focus on building the skillsets of CHWs to address the burden of hypertension within the context of LMIC. This strategy is also critical to mitigate the health care worker shortage in most LMICs.

Our findings also highlight the use of community-based multicomponent interventions to improve hypertension outcomes. These multicomponent interventions that were highlighted in this systematic review primarily focused on health education, training of CHWs, home visits, home BP monitoring, communication interventions telemedicine approaches and self-management of BP. Thus, community-based multicomponent interventions are viable strategies for addressing the growing hypertension epidemic in LMICs. As indicated by Ogedegbe et al. (2014), these multicomponent interventions are flexible to the management of hypertension at the community level which includes community screening, counseling on lifestyle modification, initiation of treatment and referral to specialist care [5]. It is therefore imperative that these interventions translate to the development and implementation of useful strategies across LMICs.

One useful strategy is the use of technology to advance health care delivery. The WHO is in support of eHealth (which refers to the cost-effective and secure use of information and communication technology in support of health and health-related sectors) such as the use of mobile wireless technologies for public health, or mHealth [46]. This review found limited use of technology at the community level to improve hypertension outcome in LMICs. In all, four studies provided interventions that used technology at the community level [26,28,32,38]. Two of the studies [32,38] that used technology were conducted in rural settings, whereas one of the studies [26] was conducted in an urban setting. These studies reported on the use of smartphones for supporting decision making as well as the provision of DVDs to improve the lifestyle of hypertension patients. There is increasing recognition of the need for using new technologies to provide an opportunity for early detection of hypertension as well as optimally control of BP levels [47]. As highlighted by Kit et al. (2019) these technologies can include a wearable wrist band to collect photoplethysmogram (PPG) and a wearable heart rate belt to collect electrocardiogram (ECG) signals [47]. Although there are calls for the breakaway from traditional cuff-based measurement of BP, the lack of accessibility and acceptability of these novel approaches in several LMICs may hinder its widespread use. The feasibility of the use of mobile technology is also a barrier, as most LMICs have limited internet connectivity. Since early diagnosis of hypertension is key to its effective management there is the need for LMICs to build the health systems capacity to make use of these novel technologies. There is a need to develop and validate such technologies that will meet the WHO criteria for use in low resource settings.

### Strengths and limitations

To the best of our knowledge, this is the first synthesis of existing literature on community-based interventions and its impact on blood pressure control in LMICs. Findings which highlight strategies that have been used in several LMIC to ensure hypertension control at the community level, can inform future evidence-based interventions. These strategies when replicated in similar settings can lead to a more optimal hypertension management in LMICs. One major limitation was our inability to assess the effect of the interventions on changes in BP due to insufficient data reporting in most of the studies. Also, the lack of useful data resulted in using twelve studies for the meta-analysis.

## Conclusion

This systematic review indicates the relevance of community-based interventions to address the burden of hypertension in LMICs. The findings highlight the need for implementing community-based strategies to ensure optimal care for individuals with hypertension. It is, however, important to evaluate how these interventions can be implemented within existing health care systems. Given the limited use of technology at the community level to improve hypertension outcomes in LMICs it is also imperative that studies are conducted focusing on feasibility, acceptability and cost of novel technologies to improve hypertension diagnosis and management at the community level.

## Additional Files

The additional files for this article can be found as follows:

10.5334/gh.1329.s1 Supplementary File 1. Supplementary Figures 1 to 3.

10.5334/gh.1329.s2 Supplementary File 2. Appendix 1. Search Strategy.

## Figures and Tables

**Table:** 

**Abbreviation**	**Meaning**
BP	Blood Pressure
CHW	Community Health Worker
cluster RCT	Cluster Randomized Controlled Trial
DBP	Diastolic Blood Pressure
JNC-7	The Seventh Report of the Joint National Committee on Prevention, Detection, Evaluation, and Treatment of High Blood Pressure
LMICs	Low- and Middle-Income Countries
MESH	Medical Subject Headings
PRISMA	Preferred Reporting Items for Systematic Reviews and Meta-Analyses
RCT	Randomized Controlled Trial
REDCap	Research Electronic Data Capture
ROBS 2	Risk of Bias 2
RR	Relative Risk
SBP	Systolic Blood Pressure
