# Supplementary material for: Community-Based Strategies to Improve Health-Related Outcomes in People Living With Hypertension in Low- and Middle-Income Countries: A Systematic Review and Meta-Analysis
Source: Glob Heart. 2024 Jun 12;19(1):51. doi: 10.5334/gh.1329 (PMC11177843; doi:10.5334/gh.1329)
Supplement: Supplementary File 1. — Supplementary Figures 1 to 3. [file gh-19-1-1329-s1.pdf]

SUPPLEMENTARY FIGURES

1	Study ID	Randomisation process	Deviations from intended interventions	Missing outcome data	Measurement of the outcome	Selection of the reported result	Overall			
2	Adeyemo et al 2013	!	+	+	-	!	-		+	Low risk
3	Lu et al 2015	+	+	+	-	+	!		!	Some concerns
4	He et al. 2017	-	!	+	+	!	!		-	High risk
5	Qi et al. 2017	+	+	+	+	!	!			
6	Sany et al., 2018	+	!	+	+	!	!			
7	Pan et al. 2018	+	!	+	+	!	!			
8	Sheilini et al., 2019	+	+	+	+	+	+			
9	Khetan et al 2019	!	+	+	-	!	-			
10	Hickey et al., 2022	+	+	+	+	!	!			

Supplementary Figure 1: Details of risk of bias assessment among randomized controlled trials

1	Study ID	Randomisation process	Deviations from intended interventions	Missing outcome data	Measurement of the outcome	Selection of the reported result	Overall			
2	Jafar et al. 2009	!	!	+	+	+	!		+	Low risk
3	Jafar et al. 2020	!	!	+	+	+	!		!	Some concerns
4	Li et al 2019	!	+	+	-	!	-		-	High risk
5	Nguyen et al 2018	!	+	+	-	+	!			
6	Vedanthan et al 2019	-	+	+	-	+	-			
7	Neupane et al., 2018	!	+	+	+	+	+			
8	Gamage et al.2020	-	!	+	!	+	-			
9	Khanal et al., 2021	+	!	+	+	!	!			
10	Suseela et al., 2022	+	+	+	+	!	!			
11	Thapa et al., 2023	+	+	!	+	!	!			

Supplementary Figure 2**:** Details of risk of bias assessment among cluster randomized controlled trials.

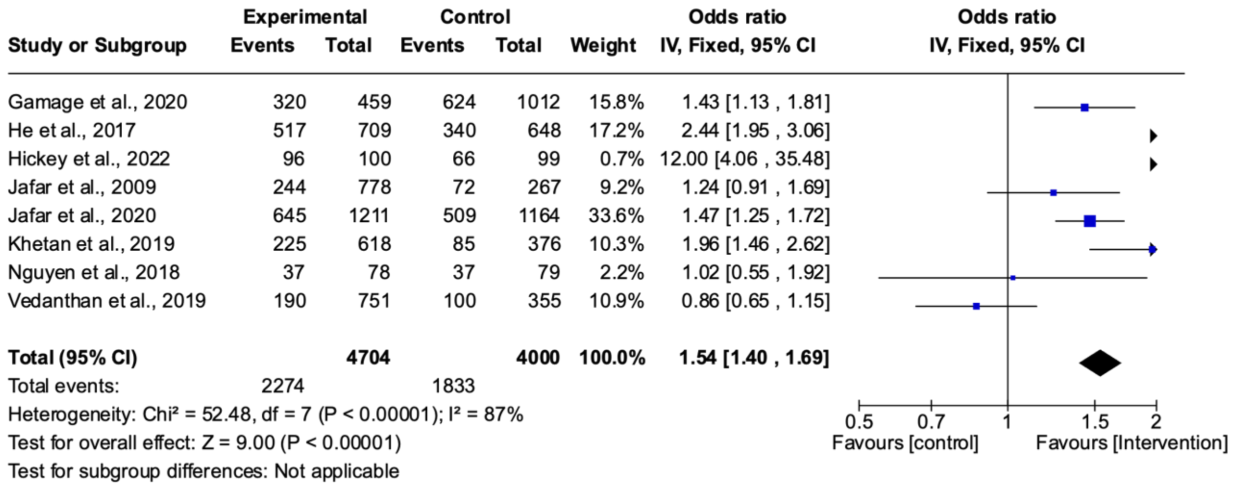

Supplementary Figure 3: Forest plot of studies that used community health workers.
