# Supplementary material for: Community-Based Strategies to Improve Health-Related Outcomes in People Living With Hypertension in Low- and Middle-Income Countries: A Systematic Review and Meta-Analysis
Source: Glob Heart. 2024 Jun 12;19(1):51. doi: 10.5334/gh.1329 (PMC11177843; doi:10.5334/gh.1329)
Supplement: Supplementary File 2. — Appendix 1. Search Strategy. [file gh-19-1-1329-s2.pdf]

Appendix 1: Search Strategy

Search Strategy for PubMed

Developing countries [MeSH] OR

Developing countr*[Title/Abstract]) OR developing nation*[Title/Abstract]) OR developing population*[Title/Abstract]) OR developing econom*[Title/Abstract] OR undeveloped countr*[Title/Abstract] OR undeveloped nation*[Title/Abstract] OR undeveloped econom*[Title/Abstract] OR undeveloped econom*[Title/Abstract] OR least developed countr*[Title/Abstract] OR least developed nation*[Title/Abstract] OR least developed econom*[Title/Abstract] OR least developed econom*[Title/Abstract] OR less-developed countr*[Title/Abstract] OR less-developed nation*[Title/Abstract] OR less-developed population*[Title/Abstract] OR less-developed population*[Title/Abstract] OR less-developed econom*[Title/Abstract] OR lesser developed countr*[Title/Abstract] OR lesser developed nation*[Title/Abstract] OR lesser developed econom*[Title/Abstract] OR lesser developed population*[Title/Abstract] OR lesser developed population*[Title/Abstract] OR lesser developed econom*[Title/Abstract] OR under-developed countr*[Title/Abstract] OR under-developed nation*[Title/Abstract] OR underdeveloped countr*[Title/Abstract] OR underdeveloped nation*[Title/Abstract] OR underdeveloped population*[Title/Abstract] OR underdeveloped econom*[Title/Abstract] OR low income countr*[Title/Abstract] OR middle income countr*[Title/Abstract] OR low income nation*[Title/Abstract] OR middle income nation*[Title/Abstract] OR low income population*[Title/Abstract] OR middle income population*[Title/Abstract] OR low income econom*[Title/Abstract] OR middle income econom*[Title/Abstract] OR lower income countr*[Title/Abstract] OR lower income nation*[Title/Abstract] OR lower income population*[Title/Abstract] OR lower income economy*[Title/Abstract] OR lower income econom*[Title/Abstract] OR resource limited*[Title/Abstract] OR low resource countr*[Title/Abstract] OR lower resource countr*[Title/Abstract] OR low resource nation*[Title/Abstract] OR low resource population*[Title/Abstract] OR low resource econom*[Title/Abstract] OR low resource econom*[Title/Abstract] OR underserved countr*[Title/Abstract] OR underserved nation*[Title/Abstract] OR underserved population*[Title/Abstract] OR underserved econom*[Title/Abstract] OR under-served countr*[Title/Abstract] OR under-served countr*[Title/Abstract] OR under-served nation*[Title/Abstract] OR under-served population*[Title/Abstract] OR under-served population*[Title/Abstract] OR under-served econom*[Title/Abstract] OR under-served econom*[Title/Abstract] OR deprived countr*[Title/Abstract] OR deprived nation*[Title/Abstract] OR deprived economy*[Title/Abstract] OR deprived econom*[Title/Abstract] OR deprived population*[Title/Abstract] OR poor countr*[Title/Abstract] OR poor nation*[Title/Abstract] OR poor population*[Title/Abstract] OR poor econom*[Title/Abstract] OR poor countr*[Title/Abstract] OR poorer countr*[Title/Abstract] OR poorer nation*[Title/Abstract] OR poorer population*[Title/Abstract] OR poorer econom*[Title/Abstract] OR lmic*[Title/Abstract] OR lmics*[Title/Abstract] OR transitional countr*[Title/Abstract] OR transitional nation*[Title/Abstract] OR transitional econom*[Title/Abstract] OR low resource setting*[Title/Abstract] OR lower resource setting*[Title/Abstract] OR middle resource setting*[Title/Abstract] OR Third world*[Title/Abstract] OR South east asia*[Title/Abstract] OR Middle east*[Title/Abstract] OR Afghanistan*[Title/Abstract] OR

Albania*[Title/Abstract] OR Algeria*[Title/Abstract] OR American samoa*[Title/Abstract] OR Angola*[Title/Abstract] OR Angolese*[Title/Abstract] OR Angolian*[Title/Abstract] OR Argentina*[Title/Abstract] OR Armenia*[Title/Abstract] OR Azerbaijan*[Title/Abstract] OR Bangladesh*[Title/Abstract] OR Belarus*[Title/Abstract] OR Belize*[Title/Abstract] OR Benin*[Title/Abstract] OR Bhutan*[Title/Abstract] OR Bolivia*[Title/Abstract] OR (Bosnia[Title/Abstract] AND Herzegovina*[Title/Abstract]) OR Botswana*[Title/Abstract] OR Brazil*[Title/Abstract] OR Bulgaria*[Title/Abstract] OR Burma*[Title/Abstract] OR Burkina Faso*[Title/Abstract] OR Burundi*[Title/Abstract] OR Cabo Verde*[Title/Abstract] OR Cambodia*[Title/Abstract] OR Cameroon*[Title/Abstract] OR Central Africa Republic*[Title/Abstract] OR Chad*[Title/Abstract] OR Comoros*[Title/Abstract] OR Congo*[Title/Abstract] OR Cote d'Ivore*[Title/Abstract] OR Cuba*[Title/Abstract] OR Djibouti*[Title/Abstract] OR Dominican Republic*[Title/Abstract] OR Ecuador*[Title/Abstract] OR East Africa*[Title/Abstract] OR Eastern Africa*[Title/Abstract] OR Egypt*[Title/Abstract] OR El Savador*[Title/Abstract] OR Equatorial Guinea*[Title/Abstract] OR Eritrea*[Title/Abstract] OR Eswatini*[Title/Abstract] OR Ethiopia*[Title/Abstract] OR Fiji*[Title/Abstract] OR Gabon*[Title/Abstract] OR The Gambia*[Title/Abstract] OR Georgia*[Title/Abstract] OR Ghana*[Title/Abstract] OR Grenada*[Title/Abstract] OR Guatemala*[Title/Abstract] OR Guinea*[Title/Abstract] OR Guinea Bissau*[Title/Abstract] OR Guyana*[Title/Abstract] OR Haiti*[Title/Abstract] OR India*[Title/Abstract] OR Indonesia*[Title/Abstract] OR Iran*[Title/Abstract] OR Iraq*[Title/Abstract] OR Jamaica*[Title/Abstract] OR Jordan*[Title/Abstract] OR Kazakhstan*[Title/Abstract] OR Kenya*[Title/Abstract] OR Kiribati*[Title/Abstract] OR Democratic People’s Republic of Korea*[Title/Abstract] OR Kosovo*[Title/Abstract] OR Kyrgyz republic*[Title/Abstract] OR Lao PDR[Title/Abstract] OR Lebanon*[Title/Abstract] OR Lesotho*[Title/Abstract] OR Liberia*[Title/Abstract] OR Libya*[Title/Abstract] OR Madagascar*[Title/Abstract] OR Malawi*[Title/Abstract] OR Malaysia*[Title/Abstract] OR Maldives*[Title/Abstract] OR Mali*[Title/Abstract] OR Marshall Islands*[Title/Abstract] OR Mauritania*[Title/Abstract] OR Mauritius*[Title/Abstract] OR Mexico*[Title/Abstract] OR Micronesia*[Title/Abstract] OR Moldova*[Title/Abstract] OR Mongolia*[Title/Abstract] OR Montenegro*[Title/Abstract] OR Morocco*[Title/Abstract] OR Mozambique*[Title/Abstract] OR Myanmar*[Title/Abstract] OR Namibia*[Title/Abstract] OR Nauru*[Title/Abstract] OR Nepal*[Title/Abstract] OR Nicaragua*[Title/Abstract] OR Niger*[Title/Abstract] OR Nigeria*[Title/Abstract] OR North Macedonia*[Title/Abstract] OR Pakistan*[Title/Abstract] OR Papua New Guinea*[Title/Abstract] OR Paraguay*[Title/Abstract] OR Peru*[Title/Abstract] OR Philippines*[Title/Abstract] OR Principe*[Title/Abstract] OR Romania*[Title/Abstract] OR Rhodesia*[Title/Abstract] OR Rwanda*[Title/Abstract] OR Samoa*[Title/Abstract] OR Sao tome*[Title/Abstract] OR Senegal*[Title/Abstract] OR Sierra Leone*[Title/Abstract] OR Solomon Islands*[Title/Abstract] OR Somalia*[Title/Abstract] OR South Africa*[Title/Abstract] OR Southern Africa*[Title/Abstract] OR South Sudan*[Title/Abstract] OR Sri Lanka*[Title/Abstract] OR Sudan*[Title/Abstract] OR Swaziland*[Title/Abstract] OR Syria*[Title/Abstract] OR Tajikistan*[Title/Abstract] OR Tanzania*[Title/Abstract] OR Togo*[Title/Abstract] OR Timor-Leste*[Title/Abstract] OR Togo*[Title/Abstract] OR Tonga*[Title/Abstract] OR Tunisia*[Title/Abstract] OR Tunis*[Title/Abstract] OR Uganda*[Title/Abstract] OR Uzbekistan*[Title/Abstract] OR Vanuatu*[Title/Abstract] OR Venezuela*[Title/Abstract] OR Vietnam*[Title/Abstract] OR (West Bank[Title/Abstract] AND Gaza*[Title/Abstract]) OR West Africa*[Title/Abstract] OR

Yemen*[Title/Abstract] OR Zaire*[Title/Abstract] OR Zambia*[Title/Abstract] OR Zimbabwe*[Title/Abstract])))

AND

Residence Characteristics [Mesh] OR Capacity building [Mesh] OR Preventive Medicine"[Majr:NoExp] OR Health Education [Mesh] OR Community Participation [Mesh] OR Community [Title/Abstract] OR Communit* [Title/Abstract] OR Community-based [Title/Abstract] OR Community-based intervention* [Title/Abstract] OR Capacity building [Title/Abstract] OR Medical management [Title/Abstract] OR Healthcare provider* [Title/Abstract] OR Preventive medicine* [Title/Abstract] OR Community involvement intervention* [Title/Abstract] OR Community action intervention* [Title/Abstract] OR Public Participation intervention* [Title/Abstract] OR Patient involved intervention* [Title/Abstract] OR Patient participated intervention* [Title/Abstract] OR Community health services [Title/Abstract]

AND

Health Services Accessibility [Mesh] OR Delivery of Health Care [Mesh] OR Health Expenditures [Mesh] OR Healthcare Accessib* [Title/Abstract] OR Healthcare Availab* [Title/Abstract] OR Entitled to Health Care [Title/Abstract] OR Receive Medical Treatment [Title/Abstract]

AND

Arterial Pressure [Mesh] OR Blood Pressure [Mesh] OR Blood pressure determination [Mesh] OR Increased Pressure [Title/Abstract] OR Diastolic Pressure [Title/Abstract] OR Systolic Pressure [Title/Abstract] OR Hypertension [Title/Abstract]

Search Strategy for Embase

'Developing countries'/exp OR

'Developing countr*':ti,ab OR 'developing nation*':ti,ab OR 'developing population*':ti,ab OR 'developing econom*':ti,ab OR 'undeveloped countr*':ti,ab OR 'undeveloped nation*':ti,ab OR 'undeveloped economy':ti,ab OR 'undeveloped economies':ti,ab OR 'least developed countr*':ti,ab OR 'least developed nation*':ti,ab OR 'least developed economy':ti,ab OR 'least developed economies':ti,ab OR 'less-developed countr*':ti,ab OR 'less-developed nation*':ti,ab OR 'less-developed population':ti,ab OR 'less-developed populations':ti,ab OR 'less-developed econom*':ti,ab OR 'lesser developed countr*':ti,ab OR 'lesser developed nation*':ti,ab OR 'lesser developed population':ti,ab OR 'lesser developed populations':ti,ab OR 'lesser developed economy':ti,ab OR 'lesser developed economies':ti,ab OR 'under-developed

countr*':ti,ab OR 'under-developed nation*':ti,ab OR 'underdeveloped countr*':ti,ab OR 'underdeveloped nation*':ti,ab OR 'underdeveloped population*':ti,ab OR 'underdeveloped econom*':ti,ab OR 'low income countr*':ti,ab OR 'middle income countr*':ti,ab OR 'low income nation*':ti,ab OR 'middle income nation*':ti,ab OR 'low income population*':ti,ab OR 'middle income population*':ti,ab OR 'low income econom*':ti,ab OR 'middle income econom*':ti,ab OR 'lower income countr*':ti,ab OR 'lower income nation*':ti,ab OR 'lower income population*':ti,ab OR ‘lower income economy’:ti,ab OR ‘lower income economies’:ti,ab OR 'resource limited':ti,ab OR 'low resource countr*':ti,ab OR 'lower resource countr*':ti,ab OR 'low resource nation*':ti,ab OR 'low resource population*':ti,ab OR ‘low resource economy’:ti,ab OR ‘low resource economies’:ti,ab OR 'underserved countr*':ti,ab OR 'underserved nation*':ti,ab OR 'underserved population*':ti,ab OR ‘underserved economy’:ti,ab OR ‘underserved economies’:ti,ab OR ‘under-served country’:ti,ab OR ‘under-served countries’:ti,ab OR ‘under-served nation’:ti,ab OR ‘under-served nations’:ti,ab OR ‘under-served population’:ti,ab OR ‘under-served populations’:ti,ab OR ‘underserved economy’:ti,ab OR ‘underserved economies’:ti,ab OR 'derived countr*':ti,ab OR ‘deprived nation’:ti,ab OR ‘deprived nations’:ti,ab OR 'derived population*':ti,ab OR ‘deprived economy’:ti,ab OR ‘deprived economies’:ti,ab OR 'poor countr*':ti,ab OR 'poor nation*':ti,ab OR 'poor population*':ti,ab OR 'poor econom*':ti,ab OR 'poorer countr*':ti,ab OR 'poorer nation*':ti,ab OR 'poorer population*':ti,ab OR 'poorer econom*':ti,ab OR lmic:ti,ab OR lmics:ti,ab OR lami:ti,ab OR 'transitional countr*':ti,ab OR ‘transitional nation’:ti,ab OR ‘transitional nations’:ti,ab OR 'transitional econom*':ti,ab OR 'transition countr*':ti,ab OR 'transition nation*':ti,ab OR 'transition econom*':ti,ab OR 'low resource setting*':ti,ab OR 'lower resource setting*':ti,ab OR 'middle resource setting*':ti,ab OR 'Third World*':ti,ab OR 'south asia'/exp OR 'southeast asia'/de OR 'borneo'/exp OR 'cambodia'/exp OR 'indonesia'/exp OR 'laos'/exp OR 'myanmar'/exp OR 'papua new guinea'/exp OR 'thailand'/exp OR 'timor-leste'/exp OR 'viet nam'/exp OR 'yemen'/exp OR 'turkey (republic)'/exp OR 'iraq'/exp OR 'africa south of the sahara'/exp OR 'egypt'/exp OR 'mauritania'/exp OR 'morocco'/exp OR 'tunisia'/exp OR 'fiji'/exp OR 'philippines'/exp OR 'samoan islands'/exp OR 'tonga'/exp OR 'vanuatu'/exp OR 'kiribati'/exp OR 'armenia'/exp OR 'ukraine'/exp OR 'bolivia'/exp OR 'el salvador'/exp OR 'guatemala'/exp OR 'honduras'/exp OR 'nicaragua'/exp OR 'haiti'/exp OR 'kosovo'/exp OR 'kyrgyzstan'/exp OR 'tajikistan'/exp OR 'uzbekistan'/exp OR 'federated states of micronesia'/exp OR 'mongolia'/exp OR 'north korea'/exp OR 'sao tome and principe'/exp OR 'solomon islands'/exp OR 'syrian arab republic'/exp OR 'palestine'/exp OR 'south east asia*':ti,ab OR 'middle east*':ti,ab OR afghan*:ti,ab OR angola*:ti,ab OR armenia*:ti,ab OR bangladesh*:ti,ab OR benin*:ti,ab OR bhutan*:ti,ab OR birma*:ti,ab OR boliv*:ti,ab OR botswan*:ti,ab OR 'burkina faso*':ti,ab OR burundi*:ti,ab OR 'cabo verde*':ti,ab OR cambod*:ti,ab OR cameroon*:ti,ab OR 'cape verd*':ti,ab OR 'central africa*':ti,ab OR chad*:ti,ab OR comoro*:ti,ab OR congo*:ti,ab OR 'cote d ivoire*':ti,ab OR djibouti*:ti,ab OR 'east africa*':ti,ab OR 'eastern africa*':ti,ab OR egypt*:ti,ab OR 'el salvador*':ti,ab OR 'equatorial guinea*':ti,ab OR eritre*:ti,ab OR ethiopia*:ti,ab OR gabon*:ti,ab OR gambia*:ti,ab OR gaza*:ti,ab OR ghan*:ti,ab OR guatemal*:ti,ab OR guinea*:ti,ab OR haiti*:ti,ab OR hondur*:ti,ab OR india*:ti,ab OR indones*:ti,ab OR 'ivory coast*':ti,ab OR kenya*:ti,ab OR kiribati*:ti,ab OR kosovo*:ti,ab OR kyrgyz*:ti,ab OR 'lao pdr*':ti,ab OR lesotho*:ti,ab OR liberia*:ti,ab OR madagascar*:ti,ab OR malaw*:ti,ab OR mali:ti,ab OR mauritan*:ti,ab OR mauriti*:ti,ab OR micronesi*:ti,ab OR mocambiqu*:ti,ab OR moldov*:ti,ab OR mongolia*:ti,ab OR morocc*:ti,ab OR

mozambiqu*:ti,ab OR myanmar*:ti,ab OR namibia*:ti,ab OR nepal*:ti,ab OR nicaragua*:ti,ab OR niger*:ti,ab OR 'northern korea*':ti,ab OR 'north korea*':ti,ab OR pakistan*:ti,ab OR palestin*:ti,ab OR 'papua new guinea*':ti,ab OR philippine*:ti,ab OR principe*:ti,ab OR 'republic of korea*':ti,ab OR rhodesia*:ti,ab OR rwanda*:ti,ab OR samoa*:ti,ab OR 'sao tome*':ti,ab OR senegal*:ti,ab OR 'sierra leone*':ti,ab OR 'solomon islands*':ti,ab OR somalia*:ti,ab OR 'south africa*':ti,ab OR 'south sudan*':ti,ab OR 'southern africa*':ti,ab OR 'sri lanka*':ti,ab OR 'sub saharan africa*':ti,ab OR 'subsaharan africa*':ti,ab OR sudan*:ti,ab OR swaziland*:ti,ab OR syria*:ti,ab OR tajikist*:ti,ab OR tanzan*:ti,ab OR timor*:ti,ab OR togo*:ti,ab OR tonga*:ti,ab OR tunis*:ti,ab OR ugand*:ti,ab OR ukrain*:ti,ab OR uzbekistan*:ti,ab OR vanuatu*:ti,ab OR vietnam*:ti,ab OR 'west africa*':ti,ab OR 'west bank*':ti,ab OR 'western africa*':ti,ab OR yemen*:ti,ab OR zaire*:ti,ab OR zambia*:ti,ab OR zimbabw*:ti,ab)

AND

Demography/exp OR ‘capacity building’/exp OR ‘preventive medicine*’/exp OR ‘health literacy’/exp OR ‘health care personnel’/exp OR ‘community participation’/exp OR

Community:ti,ab OR ‘community-based’:ti,ab OR ‘community based intervention’:ti,ab OR ‘medical management’:ti,ab OR ‘community workers’:ti,ab OR ‘community based strategy’:ti,ab OR ‘community involvement intervention*’:ti,ab OR ‘community action intervention*’:ti,ab OR ‘public participation intervention*’:ti,ab OR ‘patient participated intervention*’:ti,ab OR ‘patient involved intervention*’:ti,ab OR community health services’:ti,ab

AND

‘Health care delivery’/exp OR ‘health care cost’/exp OR ‘health care access’/exp OR ‘health care availability’/exp OR

‘Health care accessibility’:ti,ab OR ‘entitled to health care’:ti,ab OR ‘receive medical treatment’:ti,ab

AND

‘Elevated blood pressure’/exp OR

‘Increased pressure’:ti,ab OR ‘diastolic pressure’:ti,ab OR ‘systolic pressure’:ti,ab OR hypertension:ti,ab
